# Progress of the Medical Sciences

**Published:** 1901-03

**Authors:** 


### Progress of the Medical Sciences.

### MEDICINE.

**Appendicitis** still occupies a prominent place in medical literature. The frequency and serious nature of the disease no doubt justifies the place it occupies in the minds of members of the profession; but it is not to the medical or surgical aspects of appendicitis we wish to refer, but to some of the *complications* that may not have attracted general attention.

*Intestinal obstruction*, as a complication of appendicitis, is the subject of a paper by Marion.[1] He states that appendicitis may occasion intestinal obstruction in five ways: firstly, by inflammation of the intestinal wall producing paralysis of the bowel; secondly, by general adhesions of the peritoneum left after recovery from peritonitis, consequent upon appendicitis; thirdly, by localised adhesions causing fixation of a coil of intestine in an abnormal position; fourthly, by strangulation of a loop of intestine in a ring or under a band; and fifthly, a somewhat hypothetical cause is given. Marion considers that reflex disturbances may be set up by trouble in the appendix, which are sufficient to cause intestinal obstruction, and refers to cases he considers only to be explained in this way. Possibly the most interesting cause of intestinal obstruction is that given under the fourth heading. The appendix may form a ring or loop by adhesion of its extremity to the brim of the pelvis or to some point in the iliac fossa, and through this ring a coil or knuckle of intestine may slip and become strangulated. One recorded case of the kind is referred to. I happen to have met with two cases of intestinal obstruction caused by the appendix in this way. Another recorded case of somewhat different character is quoted. In that case a long appendix had become twisted round the ileum, and fixed in its encircling position by adhesion to the cæcum. The lumen of the ileum was compressed, and intestinal obstruction had followed. Most of the other cases of obstruction referred to in the paper might be produced by peritoneal adhesions following any form of inflammation of the peritoneum; but appendicitis is so common a disease, that no doubt adhesions which occasion intestinal obstruction are more commonly the result of previous trouble in the appendix than those who have not looked for this cause may be disposed to think. Marion records, amongst other cases, two examples of intestinal obstruction associated with generalised peritoneal adhesion following appendicitis, and reference is also made to various cases produced by localised adhesions, one of which is recorded by Bouglé [2] in an earlier number of the same journal.

[1] *Gaz. d. Hôp.*, 1900, lxxiii. 1439.  [2] *Ibid.*, 837.

Figures are given illustrating obstruction produced by the retention of small coils of the ileum in acutely flexed positions, and of a twist of the ileum immediately above the cæcum, which is described as a volvulus. A volvulus the result of localised adhesions would be an interesting occurrence, but it does not seem that this case was an ordinary example of volvulus. The small intestine was partly twisted round itself, but the affected coil was firmly adherent to pelvic structures by old adhesions, and the obstruction was probably due to a kink. It is, in fact, definitely stated that the coil was bent at an acute angle, and there is no mention of strangulation ot the coil such as occurs in true volvulus.

The variety of intestinal obstruction mentioned under the first heading is commonly associated with acute appendicitis, but cases may occur in which the cause of the obstruction may not be evident. Marion records a case in which neither he nor a colleague were able to arrive at a definite diagnosis until laparotomy was performed. The observation of this difficulty of diagnosis is interesting, but can scarcely be described as new. Hilton Fagge mentions that he has twice seen the abdomen opened for intestinal obstruction, and the cause found to have been appendicitis. It may be mentioned in passing that Dr. Fagge definitely speaks of inflammation of the appendix in these two cases. The morbid anatomy of appendicitis has been known to the pathologists of Guy's Hospital for many years. It is the surgery of appendicitis, rather than the pathology, which is of recent date.[1]

Another complication of appendicitis upon which a paper has recently appeared is *subphrenic abscess.*[2] Two cases are recorded—one in a woman aged forty-nine, the other in a girl aged sixteen. In the first case an attack of appendicitis had occurred five years before the time she had been seen by Dr. Baldwin. In March, 1899, she again suffered from pain in the region of the appendix. She had been losing flesh, and there was continual elevation of temperature. On July 6th the appendix was removed, but little improvement occurred, and on July 20th an abscess began to point on the right side of the abdomen near the tip of the twelfth rib. This was opened, and the pus found to come from an abscess below the diaphragm. In the second case the subphrenic abscess quickly followed gangrenous appendicitis. Dr. Baldwin states that he has found records of forty-three cases of subphrenic abscess following appendicitis, a large number of which were discovered in the *post-mortem* room. It is interesting to note that in Dr. Baldwin's cases apparently there was no abscess around the appendix which communicated directly with the abscess below the diaphragm. I have seen two cases in which a channel of pus extended from the

[1] Hilton Fagge, *Principles and Practice of Medicine*, vol. ii., 1886, p. 212. Also Wilks, *Lancet*, 1900, ii. 1303.

[2] Baldwin, *Med. News*, 1900, lxxvii. 53.

region of the appendix along the outer side of the ascending colon upwards to the liver, where it expanded into a subphrenic abscess. Such cases are, however, of much less interest than those in which no such direct connection with the appendix exists. The existence of the isolated abscess can, however, be explained on physiological and anatomical grounds. A recent paper by Dr. Byron Robinson[1] on the peritoneum refers to features of this serous membrane which explain why inflammation is so often much more marked immediately below the diaphragm than in other parts of the abdominal cavity. It may be remarked in passing that such excess of local inflammation is the rule. In both acute and chronic peritonitis the recent or organised exudation is generally much greater on the under surface of the diaphragm, over the upper surface of the liver and outer surface of the spleen, than elsewhere. It is this excessive thickening in chronic peritonitis that led to the idea that perihepatitis was a distinct and limited disease, instead of being merely a part of chronic inflammation affecting the whole of the peritoneum. The explanation of the localisation of inflammation to the upper part of the abdomen lies in "the vast function of absorption performed by the diaphragm and the very small part taken in this process by other portions of the peritoneum." The peritoneal currents run upwards, and it can readily be seen that microbes washed from the appendix to the upper surface of the liver may be delayed in their exit from the peritoneal cavity. Multiplying in their new position, they set up another focus of inflammation, which may result in suppuration, and, if adhesions be formed between the great omentum and abdominal wall, in a localised subphrenic abscess.

There is one other way in which a subphrenic abscess, secondary to appendicitis, may arise. That is through the medium of an *abscess of the liver*. In such a case it is needless to say the micro-organisms are carried to the liver by the blood. Dr. Baldwin refers to the possible existence of an abscess of the liver. I have seen such a case. An abscess, the size of an orange, opened on the upper surface of the liver, set up a subphrenic abscess, and, finally, general suppurative peritonitis. Death took place about a year after an attack of appendicitis, which was, without doubt, the cause of the hepatic abscess. In this connection it may be mentioned that a case of abscess of the liver, following appendicitis, was reported last year at a meeting of the Société de Chirurgie de Paris by M. Loison.[2] To return, however, to subphrenic abscess, Dr. Baldwin has seen such an abscess perforate the diaphragm, and discharge itself through the bronchial tubes of the right lung, with recovery of the patient. Perforation of the diaphragm is probably rare, but the spread of inflammation through the diaphragm is, no doubt, very common. In the great

[1] *Med. Rec.*, 1900, lviii. 126. [2] *Gaz. d. Hôp.*, 1900, lxxiii. 198.

majority of cases of subphrenic abscess, due to various causes, the pleura on the side of the abscess becomes involved, and the variety which follows appendicitis is not likely to be an exception to the rule. And, apparently, in some cases the pleural effusion may be so extensive, and the amount of pus below the diaphragm so slight, that the condition of the chest is the most prominent feature of the illness. M. Dieulafoy has written a paper upon this subject, and describes the condition as "*pleurésie appendiculaire*."[1] Two cases are described which came under his own observation, and others are referred to recorded by several writers. Usually the pleural effusion is said to be large in amount and offensive, and in some cases a tympanitic note at the upper part of the chest may show the presence of gas; in other words, there may be a pyo-pneumothrax. It is scarcely necessary to add that the effusion is on the right side of the chest. A brief outline of one of the cases of M. Dieulafoy may be of interest. A man, aged 26, was admitted to l'Hôtel Dieu with a pleural effusion obviously filling almost the whole of the right side of the chest; a tympanitic note being present only at the apex. The patient presented not only the evidence of dyspnœa, but was collapsed. M. Dieulafoy therefore lost no time in ascertaining the nature of the fluid in the chest, and a syringeful of turbid offensive fluid was drawn off. Before, however, an operation for the evacuation of the fluid could be performed the patient died. Apparently nineteen days before admission he had commenced to suffer from pains in the lower part of the right side of the abdomen, which four days later had spread to the hepatic region. Pains in the right side of the chest followed, and the medical man in attendance called a *confrère* into consultation, who found a pleural effusion at the back of the chest and a loud pleuritic rub in front. As already indicated, nineteen days after the onset of abdominal pain he was removed to the Hôtel Dieu, and there died before anything definite for his relief had been attempted. At the autopsy, in the right pleural cavity were found over six pints of fœtid pus. Channels of pus were found passing up behind the cæcum and colon to the upper surface of the liver, but apparently the suppuration in the abdominal cavity was not great. Only a thin layer of pus covered the right lobe of the liver. Old peritoneal adhesions were present, but no recent general peritonitis. The appendix was inflamed and almost gangrenous, but no perforation had taken place. In the above case the progressive advance of the inflammation from the region of the appendix to the upper surface of the liver, and from there to the right pleura, is obvious from the history; and such a history, when one's attention has been drawn to this possible sequence of events in appendicitis, would suggest the correct diagnosis. Yet the discovery of fluid in the chest may monopolise attention,

[1] *Bull. Acad. de. Méd.*, 1900, 3e Sér., xliii. 438.

and its sub-diaphragmatic origin be unsuspected. Even where a subphrenic abscess has been present, Dr. Baldwin remarks that the presence of symptoms and physical signs pointing to the chest has led to the suppuration below the diaphragm being overlooked during life; its presence having been only discovered at the autopsy.

Dieulafoy has described pleural effusion following appendicitis as "*pleurésie appendiculaire*," and the name implies that the pleurisy is a complication of appendicitis, but *pneumonie à forme appendiculaire* is a name given to pneumonia when the symptoms resemble those of appendicitis. In a recent article upon this subject,[1] Morris, Mirande, and Hutinel are mentioned as having described cases. The disease is said to commence with the classic symptoms of appendicitis, which last three or four days. There is repeated shivering, with vomiting, and pain in the right iliac fossa. Cases have been operated upon, and the appendix found to be perfectly normal. Apparently not until the fourth day are there symptoms and physical signs which attract attention to the chest. Mistakes are said to be most likely to occur in children, but difficulty of diagnosis may also be present in adults. We are all aware how cases of familiar disease may sometimes prove to be difficult of recognition, yet one cannot help thinking that the mistake of diagnosing appendicitis where pneumonia exists must be in great measure due to the large place appendicitis occupies at the present time, in the minds of the medical profession. Physical signs of lobar pneumonia in children are often very late in appearing, in fact one may say that it is the exception rather than the rule to find evidence of the presence of consolidation before the fourth day of the disease. Abdominal pain and vomiting also are not uncommon; but an abnormal rapidity of breathing, if looked for, generally correctly suggests to the observer the nature of the complaint, which will definitely manifest itself at a later date. Yet in the light of records of other cases referred to above, which show that inflammation of the pleura may accompany appendicitis, it is clear that one must be careful not to go to the other extreme and ignore symptoms that suggest trouble in the abdomen.

THEODORE FISHER.

### SURGERY.

Reports of cases of **actinomycosis** are disquieting reading, for there can be no doubt that the disease is still regarded by many as rare and infrequent, whereas records show that this view is mistaken, while it is abundantly evident that there is very serious danger—in spite of being alert and on the watch—of

[1] *Semaine méd.*, Janvier 9, 1901, p. 13.

failing to recognise the condition. This arises from the obscure and ill-defined symptomatology of the disease, and its close resemblance in clinical features to several much commoner lesions.

These difficulties have been emphasised by Mr. Godlee[1] in a recent account of thirteen cases. The specific organism—streptothrix actinomyces—grows on cereals and grasses, and may apparently be communicated in the dust from a threshing machine; by chewing fresh corn, or sucking straw or grass; possibly directly from an infected animal; and very likely in many other ways. The incubation period is said to vary from a week to several years. Once implanted, the organism grows and sets up inflammation. Sometimes an ulcer is formed; at other times a tumour, which is "of a pale yellowish colour, globular in shape, riddled with holes of a larger or smaller size containing pus, and very vascular, although it presents a superficial resemblance to the interior of a tuberculous or gummatous deposit."[2] It infiltrates all the tissues, spreading in the intermuscular planes, and even into the muscles, entering into the cancellous tissues of bone, and the solid viscera, and sometimes forming communications between the hollow viscera. The growth may be carried by the vascular channels as emboli, and deposited in distant parts of the body.

Pathologically, the tumours consist of a network of fibrous tissue, whose interstices are filled with masses of streptothrix surrounded by a zone of small round and epithelioid cells, and occasional giant cells. Sometimes the fibrous network is excessive, and a more definite tumour results, or it may so much increase as to crowd out the organism, producing a spontaneous cure.[3] Much more frequently there is an over-production of the streptothrix with breaking down of the tissue and formation of an abscess, with a resulting sinus when it opens or is incised. It is in the discharge from such an abscess or sinus (as also in the expectoration, or in the stools in some cases) that the first definite indication of the disease is to be sought. These are the so-called "sulphur granules"—small round granules, sometimes very minute, sometimes larger; generally pale yellow, but sometimes white—which are demonstrated by allowing the discharge to run over a microscopic slide, or down the side of a test-tube. According to Dr. Ruhräh,[4] Langhans has reported a case in which the granules were black, and he says they may be of a greenish hue. If one of these granules be placed in a little water on a slide, and gently pressed under a cover-glass and examined under a high power, "a densely felted mass of threads" will be seen with the so-called clubs arranged radially around it.[5] In some cases, however, the latter are not to be seen. The disease appears to be more than twice as common

[1] *Lancet*, 1901, i. 3. [2] *Ibid.*
[3] J. Ruhräh: "Actinomycosis in Man," *Ann. Surg.*, 1899, xxx. 429.
[4] *Loc. cit.*, 425. [5] Godlee, *loc. cit.*

in men as in women, and to occur most frequently during the third decade of life.

By combining statistics of various observers, including a large number of cases, Dr. Ruhräh finds the percentage for different regions to be as follows: head and neck, 55 per cent.; digestive tract, 19 per cent.; pulmonary, 14 per cent.; skin, 2 per cent.; and doubtful, 5 per cent. If the recorded cases are examined it will be seen how difficult the diagnosis often is, and how close the likeness sometimes is with tubercular affections and chronic inflammations especially, and not infrequently with syphilitic lesions—chiefly gummata—and even new growths.

It will be convenient to consider the cases under the above general divisions:—

*Head and Neck.* (1) Actinomycosis of the jaw. This generally simulates the common periosteal or alveolar inflammations, or occasionally suppuration in the antrum. The chief characteristics appear to be that one swelling follows another with great inveteracy, sinuses are very obstinate, and induration, or infiltration, is apt to occur widely in either an upward or downward direction.

Dr. Ruhräh[1] considers that the cases where the lower jaw and adjacent structures are involved (temporo-maxillary) have a more distinct clinical history than most other forms, the symptoms being an almost constant sharp pain on a level with the last molars, the early appearance of *trismus* of varying intensity, and the boggy consistence of the swelling. He also lays great emphasis on the absence of involvement of the lymphatics, which he describes as a constant feature of the disease, no matter where the site may be. The trismus is attributed to an involvement of the muscles of mastication, although in some cases the temporo-maxillary joint is affected. Carious teeth are usual, but not, judging by the records, invariably present. In a case under the care of Dr. C. A. Hoover[2] it had never been possible during life to demonstrate the streptothrix, although the patient was under observation for many months, with repeated operative treatment; and at the autopsy very widespread disease was found.

(2) The disease may appear as an ulceration of the mucous membrane of the mouth, as in an interesting case published by Dr. J. C. Oliver.[3] A sore developed at the left angle of the mouth three or four years before, which was papillomatous, with a slight ulceration around. This was curetted and healed, leaving a "white indurated patch." Two similar sores developed later, and were treated in the same way, with the same result. Coincidently a purulent affection of the gums appeared, for which all the lower teeth, except the last two molars, were

[1] *Loc. cit.*, 436. [2] Quoted by Ruhräh, *loc. cit.*, 438.
[3] *Ann. Surg.*, 1900, xxxii. 668.

extracted, and the artificial teeth were clamped to these teeth. An ulcer appeared opposite the clamp in the right cheek, and spread rapidly. No streptothrix or tubercle bacilli were found in the scrapings. Iodide of potassium was then given in large doses for two months, on the chance of the lesion being syphilitic, but the ulceration only extended farther and became very painful. Swellings formed in the submaxillary region and at the angle of the jaw, and the condition suggested epithelioma. While an operation for this was being considered the submaxillary swelling softened. On incising this "a thin, greenish-yellow fluid containing some yellowish-white curdy masses" was discharged. The microscope again failed to show the streptothrix. Eventually a radical operation was undertaken, but within three weeks an enlargement began just behind the scar of the incision in the neck; œdema of the face, and signs of antral disease, followed, and finally brawniness of the right side of the neck, with four or five lumpy enlargements under the jaw. A lump under the ear softened, and on being opened the same greenish fluid was evacuated with "white or whitish-yellow particles, looking like caseous masses," among which, after prolonged search, the streptothrix was discovered. The patient eventually died of arterial hemorrhage, probably from the lingual artery.

(3) Actinomycosis about the gum seems to be often manifested by fungous masses of granulation tissue about a carious tooth.

*Pulmonary.*—This form may apparently start as a broncho-pneumonia, or pleuro-pneumonia where the disease has spread from adjacent structures, but the two kinds assimilate in the later stages. Many cases have been recorded of this class of disease, and one or two features are conspicuous in most of them. Almost invariably we find the appearance early in the disease of abscess-swellings over some part of the chest wall, or under the costal edge—much more occasionally a pleurisy or empyema—with many of the signs of tubercular disease of the lungs. In this latter connection Israel[1] stated that these signs were almost always in the *lower* lobes of the lung, but there would seem to be many exceptions to this rule. Fever is an early symptom, and resembles the tubercular type, but it depends a good deal upon the development of secondary infections which are very common. There is always expectoration, which is often fœtid, and occasionally bloody; but hæmoptysis is very rare. The organism can generally be found in the expectoration, although it is sometimes apparently absent. A constant symptom is pain referred to the lung of the same side. A lateral curvature of the spine often occurs, the concavity being towards the affected side.

*Abdominal.*—The two most practically important varieties under this heading are actinomycosis affecting the liver, and

[1] Quoted by Ruhräh, *loc. cit.*, 608.

some part of the alimentary tract. (1) Dr. Ruhräh states that the disease is never primary in the liver, whereas Mr. Godlee[1] reports six cases in which he believed that organ to have been first infected. In all cases lung or thoracic symptoms became prominent features, and in several no enlargement of the liver was felt below the costal edge. One case[2] was very interesting, as showing the extreme diffusion by emboli that may occur; the interest of another[3] lay in the failure, in spite of repeated examinations, to detect the fungus, which was due to the mycelium being more delicate than usual, and not presenting the clubs. The same kind of lateral curvature as seen in the pulmonary form is apt to occur in these cases. (2) In the alimentary tract infection occurs where the intestinal movements are most sluggish, and the cæcum and its surroundings—especially the appendix—is the favourite site. In 40 cases collected by Grill,[4] in which the point of entry was definitely made out, 18 began here. Hinglais[5] found this region implicated in 60 out of 100 abdominal cases. He describes the disease here as having four stages: (1) Intestinal disturbance, diarrhœa persistent, with perhaps blood and mucus, and tenesmus; rarely constipation. There is no tumour to be made out by palpation or rectal examination. (2) Localised pain, with a tumour of unequal resistance—the skin becoming reddened, and later violet-blue. This stage may last a varying time, and there may be remissions. The disease may now extend to other organs. It may become infected with other organisms and extensive abscess formation take place, most frequently ending in (3) the formation of fistulæ. (4) The disease may be arrested and a spontaneous cure result. Cases, however, will be found recorded with a much less definite course.

*Skin.*—These cases are quite rare, but Leser[6] describes two forms—(*a*) ulceration; (*b*) a discrete nodular skin inflammation, with central cicatrisation and peripheral extension, as in lupus. He considers the absence of lymphatic involvement almost pathognomonic, and he says the board-like indurations and the grossly nodular conditions which are found in these cases are not usual in the other affections. Actinomycosis of the skin may also be mistaken for rodent ulcer.

The **diagnosis** of actinomycosis has been partly considered in the foregoing remarks, but a few special points may be brought into prominence here. The only absolutely diagnostic feature is the discovery of "sulphur granules," in the discharge from sinuses or abscesses, or in the sputum or stools; but there are other indications that are very suggestive, and should arouse suspicion. Great stress has been laid by some observers

[1] *Loc. cit.* [2] Case VI., p. 6. [3] Case VII., p. 7.
[4] Quoted by Ruhräh, *loc. cit.*, 620.
[5] Quoted by Ruhräh, *loc. cit.*, 622. [6] Quoted by Ruhräh, *loc. cit.*, 722.

on the absence of involvement of the neighbouring lymphatics in all cases. The character of the abscess, as determined by the finger, Mr. Godlee[1] says would make him almost certain of its nature—"the indefinite spongy walls, easily breaking down before the finger in all directions and bleeding very freely, are almost unmistakable when they have been felt two or three times." Dr. Ruhräh,[2] in commenting on a case of Dr. Blake's which he saw, says the back was the seat of numerous sinuses, "whose puffed and reddened openings, together with the livid intervening skin, gave a perfect picture of actinomycosis"; and Volkmann, Boström, and Bernhardt[3] believe that the reddening of the skin turning later to a violet-blue tint, shading from the centre to the periphery, is "diagnostic" of the disease. Under the term pseudo-actinomycosis are grouped diseases which resemble in their lesions actinomycosis as it has been described here, and which are supposed to be caused by separate organisms. How far this view will be borne out time must decide; meanwhile it may be noted that it has been suggested that streptothrix actinomyces and bacillus tuberculosis may be closely allied organisms, in which view the clinical association of their lesions is significant.

The **prognosis** seems to depend (1) on the possibility of a radical extirpation of the growth—that is, on its size and the position it occupies; (2) on the presence or absence of secondary infection, as in the former case metastases are said to be more apt to occur, and these are the conditions in which the administration of the iodides often fails; they are also more likely to be extensive.

**Treatment.**—This is indicated by what has gone before. Whenever possible the diseased area should be completely excised, and Dr. Ruhräh recommends that after this has been done the area (when it is permissible) should be thoroughly cauterised. The administration of iodide of potassium should always be pushed at the same time, beginning with 5 or 10 grains three times a day, and increasing it to 40 or 50 grains thrice daily. In the pulmonary cases it is recommended to use oil of eucalyptus both internally and by inhalation, but this should not interfere with the exhibition of iodide of potassium. For injections into sinuses, etc., a number of substances have been employed—tincture of iodine, carbolic acid, salicylic acid, nitrate of silver solutions, iodoform, etc., etc. But the only hopeful treatment seems to consist in excisions, or curettings, repeated as often as necessary and as early as possible, with the internal administration of iodide of potassium up to the limits of the patient's endurance.

W. M. Barclay.

[1] *Loc. cit.*, 6. [2] *Loc. cit.*, 615.
[3] Quoted by Ruhräh, *loc. cit.*, 622.

### OPHTHALMOLOGY.

The special attention which has been drawn towards our army during the past year, and the suggestions for reform, have given fresh interest and importance to the discussions held at the Annual Meeting of the British Medical Association, at Portsmouth, as to the standard of vision required, and as to the **visual tests** used in examining candidates for the army, navy, and mercantile marine.

The statement made that the Boer can see twice as far as the British soldier has excited much comment, and emphasised the necessity for good eyesight in soldiers, particularly under the conditions of modern warfare. In our army—although the large majority of our soldiers may see well — we are probably admitting a considerable number of men with such defective eyesight as to render them not only useless for purposes of attack, but positively dangerous to their own comrades when important duties, such as sentry work or scouting, have to be performed.

Deputy-Surgeon-General Cayley, I.M.S., in opening the discussion, said: "It would of course be most desirable that all the men in the navy, the army, and other services should have normal vision, but such a standard seems to be quite unattainable. To get the requisite number of recruits, the services are forced to accept men whose vision is up to a certain standard of practical fitness, though below normal."

Recruits to the British army are required to be able to count round black dots $\frac{1}{5}$th inch in diameter at a distance of 16 feet. This corresponds with the ability to see the 3-foot bull's-eye target at a distance of 600 yards, or to read Snellen's type letters D=24 at 6 metres ($\frac{6}{24}$). Sir T. Longmore found that this standard of vision is represented by a myopia of 1.75 D. A man with 1.75 D of myopia has no clear vision beyond about 3 feet. He does not recognise a man's face beyond 6 yards, instead of at about 80 yards; he cannot see a man more than 400 yards, instead of 1200 yards from him, nor a mounted moving man more than 700 yards away, instead of 2000, as could be done by a person with normal sight. A soldier with such imperfect vision cannot carry out in a reliable manner the duties required of him under the conditions of recent warfare.

The standard of vision required by officers in all branches of the army is at least $\frac{6}{24}$ on Snellen's method, with each eye separately, unaided by a lens; and, in addition, with suitable lenses, one eye must have full vision and the other $\frac{6}{12}$. Any myopia that is present at the age when officers join the service is likely to develop still further, and in the writer's opinion the necessity for the use of glasses is, if possible, to be avoided, and without their assistance the standard required is not high enough. It could be raised to $\frac{6}{18}$ or even to $\frac{6}{12}$ without any material loss of suitable candidates. The small number of marks which separate the successful from the unsuccessful competitor in the literary examination would be often more than compensated for by a definite superiority in eyesight or physique. Plenty of young men are likely to continue to apply for commissions whatever regulations are required, and a severe examination of physique and eyesight should precede the intellectual one. In the case of private soldiers we may have to be content with a low standard of eyesight, as of other qualifications; but a large majority of those wishing to enlist would be found able to pass a much more stringent examination than is now required, and it is questionable whether it is advisable to admit combatants who fall short in physique or in eyesight, in order to increase the numerical strength of our forces, to the detriment of their value as a fighting machine. Certain branches of the service absolutely require perfect eyesight—the cavalry man, who has to act as scout; the foot soldier, who has to do sentry work or to fire at an enemy 1,000 or 2,000 yards away; and especially the artilleryman, who is responsible for the laying of a field-piece. For engineers and those concerned in office routine, and for branches requiring more mental effort or closer handiwork, a degree of short-sight otherwise prohibitive may be allowed.

Not only is the standard of vision too low, but the present method of testing eyesight for the services is unsatisfactory. 1. The standard of vision is an uncertain quantity; it varies in degree according to the light used, and with the particular material on which the letters are printed. There is much difference between the illumination in a dark or in a light room, and between the latter and open daylight; while the daylight may constantly vary. A definite standard of illumination should be employed if a mathematical standard of vision is to be enforced. Many myopes are able by a strained effort of the orbicularis, or with the finger, to produce a contraction of the palpebral aperture, which materially improves the sight, and no objection appears to be raised against this artificial aid, without which the candidate would in many cases fail to see the selected types. 2. The testing is entirely subjective, and no attempt is made to estimate the real condition or refraction of the eye. A candidate may pass the tests readily, and yet have such a degree of hypermetropia that glasses will soon be required. As Dr. George Mackay said in a paper on "Eyesight and the Public Services" before the Ophthalmological Society, on January 31st, 1895: "I am certain that the present neglect of refraction in estimating visual capacity is costly to the employer (whether the State, the company, or the capitalist), and the cause of hardship to the employed;"[1] and the opinion

[1] *Tr. Ophth. Soc. U.K.*, 1895, xv. 201.

of those best qualified to judge on this matter was expressed in a resolution passed at the same meeting to the effect that "no examination of eyesight is fair or satisfactory which depends merely upon the examination of the form-vision by test types and dots, and omits an estimation of the refraction." It is urged by those who are responsible for the examination of recruits that the time required for objective testing of the refractions in each case would be too long; but any ophthalmic surgeon could almost as rapidly decide whether the ametropia was outside a given amount, as whether the candidate possessed the necessary standard of vision for counting dots.

Candidates for commissions in the royal navy are not considered eligible who are subjects of any degrees of myopia or hypermetropia. For admission to the navy perfect form and perfect **colour vision** are required. A limited discretionary margin is allowed in the case of certain classes of officers, such as chaplains and clerks. Some officers of the navy have short-sight, which had not declared its presence at the early age when they entered the service. If it is essential that seamen in the royal navy should have perfect vision, those in the mercantile marine, and particularly those employed upon the passenger liners, should also be required to have good sight. The Board of Trade regulations on the subject cannot be considered satisfactory. Ordinary seamen are not required to pass any visual test, although they may be called upon to act as look-outs, and thus have the safety of a whole ship dependent upon their good sight. Certificated officers and apprentices, however, are required to have perfect colour vision as tested by Holmgren's wools, and with either one or both eyes open must possess $\frac{1}{3}$rd to $\frac{2}{5}$ths of normal form vision. This standard is too low, particularly when we consider that the test is conducted by lay examiners, whose observations or deductions may not be perfectly reliable. Moreover, Bickerton has stated that even after a man has failed to pass the Board of Trade tests for form or colour vision, and has been officially announced to be nautically blind, there is no provision made to prevent him from continuing to follow his occupation as a seaman.

A pilot has the entire responsibility for the steering of a ship from the moment he takes charge of her. The authorities evidently recognise that pilots should have good sight, as they require both perfect form and colour vision from those who are employed in the Indian pilot service. In the British Isles, however, no standard of vision for pilots has been formulated, nor is their sight tested, except in accordance with the local regulations at a few ports.

For seamen and railway employés perfect colour vision is of special importance, and a thorough application of Holmgren's wool test, now officially recognised by the Board of Trade and in all branches of the public services, would appear to be a reliable means for judging of the colour sense.

It would seem, however, to be possible that a candidate who has, without hesitation, matched all the skeins correctly may, under the actual conditions obtaining at sea, be unable to estimate correctly his light signals; and Bickerton[1] quotes, among other cases, one in which the mate of a 4,000-ton steamer, who could match Holmgren's wools perfectly, mistook the green side-light of a ship for a white masthead-light, and almost caused a collision owing to his error.

With a view of detecting such cases of colour defect (which he thinks may depend on scotomata), Grossmann[2] has devised a new apparatus by means of which colour effects can be compared under varying intensities of degree or illumination. He describes this instrument as follows: "In order to reproduce in the apparatus the actual conditions of practical life, I took as the source of light an ordinary signalling hand-lamp, such as is used on some railways. In front of the bull's-eye are placed two metal discs, capable of being moved round their centres, and perforated near their edges by circular apertures filled with glasses of various colours. The colours in each of the discs correspond, though arranged in different order. On the other side of the discs is a metal plate which overlaps them, and is perforated by two holes, one beside the other. Each of these holes is so placed, that by rotating the disc on its corresponding side, each of the coloured glasses is in turn brought between it and the bull's-eye of the lantern.

"Between the bull's-eye and each opening in the plate there is an iris diaphragm, with indicators showing the diameter of the apertures in millimetres. Between the diaphragms and the lamp two slips of glass, suitably mounted, are made to slide up and down. These slips are tinted neutral grey, the intensity being graduated from complete transparency at one end to almost complete opacity at the other."

By this means the opening for the light falling through a coloured glass can be made to vary in any size from 10 mm. diameter to pin-point, and in intensity from light to dark. An almost unlimited variety of lights, both coloured and "white," can thus be obtained. Both discs can be moved independently, as can also the diaphragms and glass slips.

A convenient way of using the instrument is to place it facing a mirror 15 feet away, from which the coloured lights can be seen reflected. Having suitably adjusted the iris diaphragms and tint slips, select a colour on one disc, and direct the "patient" to turn the other disc around until the colours as seen in the mirror match; or a colour may be exposed on one disc, and then covered over while the patient tries to match it on the other disc from memory.

[1] *Brit. M. J.*, 1900, i. 621. [2] *Ibid.*, 1900, ii. 1764.

The method is more simple than might appear from this description of it; and coloured lights can be examined through it nearly under the conditions obtaining in actual life, both by rail and sea. Though much care is already taken, no efforts should be spared to still further diminish the danger of accidents being caused by imperfect eyesight.

F. R. Cross.

---

### Reviews of Books.

